# Career Choices of Medical doctors at Graduate level - A Multicenter Study

**DOI:** 10.12669/pjms.335.12945

**Published:** 2017

**Authors:** Sadaf Zia, Maisam Abbas, Mehreen Sulaiman, Salman Matiullah Sheikh

**Affiliations:** 1 Sadaf Zia, FCPS. Assistant Professor, Department of ENT, Dow International Medical College Dow University of Health Sciences, Karachi, Pakistan; 2 Maisam Abbas, FCPS. Assistant Professor, Department of ENT, Jinnah Medical & Dental College, Karachi, Pakistan; 3 Mehreen Sulaiman, MBBS. House Officer / Intern, Department of ENT, Dow International Medical College Dow University of Health Sciences, Karachi, Pakistan; 4 Salman Matiullah Sheikh, FCPS. Professor and Head of Department, Jinnah Medical & Dental College, Karachi, Pakistan

**Keywords:** Career choices, Medical education, Medical students

## Abstract

**Objective:**

To find out the specialty choices being taken by Final year Medical students and graduate Doctors.

**Method:**

This is a cross sectional survey study which was conducted over two months from 1^st^ November to 31^st^ December 2016. Final year students and house job doctors were asked for the filling of Performa, after filling the consent form. A self-developed, anonymous questionnaire was used to conduct the study using close ended type of questions. This was a multi-center study conducted at Dow International Medical College and Jinnah Medical and Dental College. An IRB approval was taken for the study. A total of 317 individuals completed the Performa. Demaographic data included information regarding the year of passing, number of family members already in the medical profession, then specific questions were asked regarding their future career choice and the reason for choosing that particular speciality. After collection of data from both the centers a single operator entered the Data on SPSS 16 version. Frequencies and chi-square test were performed and p-valves were tabulated.

**Results:**

A total of 317 individuals completed the Performa. Two hundred and nine participants (65.9%) were females and one hundred and eight (34.1%) were male participants. The age ranged from 22-29 years mean of 25.15 and SD of 1.348. One hundred and twenty one (38%) had a family member as a doctor in the family. Medicine and allied was the most sought after specialty 184(58%), followed by surgery and allied in 108(34%). Non-Clinical Specialty such as radiology, basic sciences was taken up by 27(7.9%).

**Conclusion:**

The working hours followed by passion for the chosen field were the important reasons for selecting any specialty. The next most important reason was higher income and other family responsibilities of an individual. The ladies are opting more for fields with a controllable life style.

## INTRODUCTION

In the final year of medical school and immediately afterwards MBBS, the fresh graduates usually plan for the career ahead they are likely to follow in their life.^1^,^2^ For some it is a straightforward decision whereas for others it is a dilemma to choose what path to pursue.^3^ Usually after MBBS a fresh graduate has multiple different paths and careers to choose from ranging from clinical sciences, applied medical sciences, pharmaceutical, information technology and other non-clinical fields.^4^ These scopes are open to the fresh graduates not only in the country but abroad as well. The choice of a particular field to pursue rests on many factors; from social, psychological, peers to family and financial factors.^5^

Around the globe we see different trends for medical students choosing a particular set of career choices after completing their bachelor degree with various reasons pertaining to their decisions.^6^ In the neighboring country; a study quoted that a majority of students preferred to choose a post graduate career in medicine and pediatrics followed by surgery.^2^ Students stated as financial benefits and private practice was the most common reason for opting these specialties.^4^,^5^

A large number of female medical students do not pursue their career after graduation for a variety of reasons such as managing a family, society status as doctor in order to get married and certain fields which are seen as ‘male - dominated’ further contributing to this anarchy, uncertainty of fields and loss of human resource. A sizeable chunk of male doctors also depart into non-medical and commerce related fields after completing their bachelors; the reasons being less challenging and more economically stability at an earlier age.^2^

Medical graduate’s choice of specialty also relies heavily on which field is presented to them as attractive and their rotation in the different specialties.^6^ Without a proper career counseling and knowledge of respective available fields to these fresh doctors, their choices can be restricted to certain specialties resulting in shortcomings in other disciplines for trainee doctors.^7^,^8^ This would also lead to saturation in one area of sub-specialty with shortage in another resulting in disproportionate balance of human resources.

Our objective was to document the specialty choices being taken by Final year Medical students and graduate Doctors. This data can be utilized to promote education among medical students regarding other less opted specialties and to properly and efficiently utilize this data to create job opportunities and similarly distribution of postgraduate slots in all specialities.

## METHODS

This is a cross sectional survey study which was conducted over two months from 1^st^ November to 31^st^ December 2016. Final year students and house job doctors were asked for the filling of Performa, after filling the consent form. A self-developed, anonymous questionnaire was used to conduct the study using close ended type of questions. This is a multi-centre study conducted at Dow International Medical College and Jinnah Medical and Dental College, Karachi.

An IRB approval was taken for the study with reference no. JMC.ERRC.2810.16 dated October 28, 2016. The sample size was calculated by Open Epi calculator. With 6% margin of error and 95% confidence level, the total sample size came out to be 267.^9^ Convenience sampling will be used. A total of 317 individuals completed the Performa. Demaographic data included information regarding the year of passing, number of family members already in the medical profession, then specific questions were asked regarding their future career choice and the reason for choosing that particular specialty. After collection of data from both the centers a single operator entered the Data on SPSS 16 version. Frequencies and chi-square test were performed and p-valves were tabulated.

## RESULTS

A total of 317 individuals completed the Performa. Two hundred and nine participants (65.9%) were females and one hundred and eight (34.1%) were male participants. one hundred and thirty eight (43.5%) participants were in the final year of MBBS and one hundred and seventy nine (56.6%) were doing their House job/Internship.

The age ranged from 22-29 years with mean of 25.15 and SD of 1.348. The female to male ratio is 2:1. Two hundred and seventy seven (87.3%) belonged to government teaching institute and 40(12.6%) belonged to private medical teaching institutes. Two hundred and sixty three (82.9%) had a Pakistani origin and 54(17%) were foreigners with Pakistani origin.

**Fig.1:**
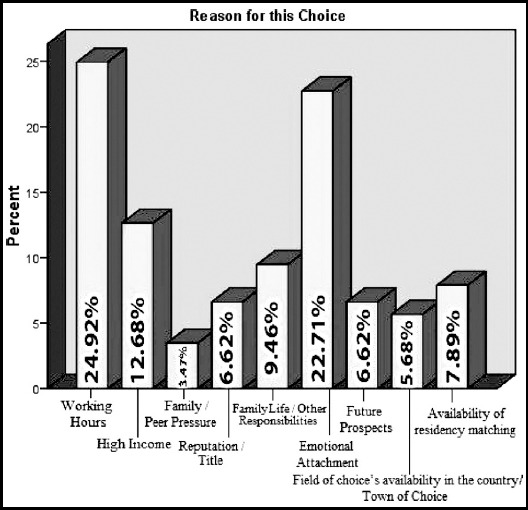
First Priority Reason for selecting a specialty.

**Fig.2:**
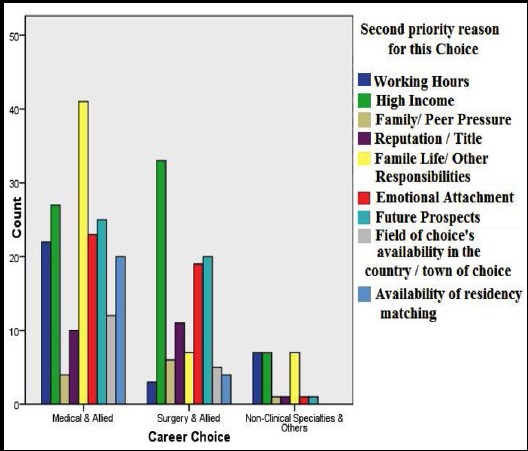
Second important Reason for selecting a specialty.

One hundred and twenty one (38%) had a family member as a doctor in the family. Except five most of them had more than one family member as a doctor. Medicine and allied was the most sought after specialty 184(58%), followed by surgery and allied in 108(34%). Non-Clinical Specialty such as radiology, basic sciences was taken up by 27(7.9%). When asked about the reasons for selecting their chosen specialty 24.9% reported it as the number of working hours and emotional attachment as the most important reason of their choice followed by high income and other family responsibilities. Fig.1 and 2.

When the specialty choices were compared with the country of origin the overall *p-value* was 0.006. This was again highlighted when the choice of surgery and allied was separately compared with the country of origin; the p-valve was 0.005 which is significant. The non-clinical specialty also had a strong association with the country of origin; the p-valve was 0.000 Table-I. The presence of a family member as a doctor also had a significant co-relation with the career choices taken with an overall p-valve of 0.016, The final year students and House job/ Internship participants were compared for their choices for the three groups but their p-value was not found to be significant.

**Table-I:** Comparison of specialty choices.

*n=317*		*Medicine & Allied*	*Surgery & Allied*	*Non-Clinical*
Comparison of Specialty Choices- p-values	Country of Origin	1.000	0.005	0.000
	Doctor in family member	0.454	0.204	0.200
Comparison of Male & Female Doctor Choices for Careers	Male	63	39	06
	Female	121	69	19
	P-value	0.299	0.008	0.619

**Fig.3:**
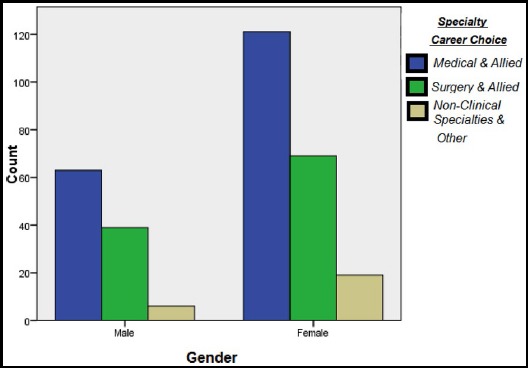
Gender wise distribution of specialty choices.

For those individuals who choose Medical and allied the reason of their choice was the consideration of working hours followed by the quality of family life or family responsibilities. The female participants were taking such fields more as compared to male participants, Fig.3. In surgery and allied the individuals choosing this specialty the most important reason for them was emotional attachment followed by the higher income attraction. The reasons for the non-clinical specialty were the same as those for medicine and allied.

## DISCUSSION

Our study showed higher number of females going for professional education with female to male ration of 2:1 which is the reverse of what it used to be 15-20 years ago in Pakistan.^9^ Professional education had been male dominant is the previous years a trend which is changed in the recent articles.^10^ The reason for this trend could be the open merit system adopted in Pakistan in 1991, there are other reasons of the brain drain as well.^11^,^12^ Internationally as well the increasing number of females in different specialities has been noted.^13^ An article published in 2014 also sheds some light in the migration abroad of trained doctors contributing to this shortage. It is also stated that various reasons from financial to current law and order situation of the country are contributing to this ‘brain drain’. Thus these doctors are sometimes forced to choose the specialty available in order to go abroad and therefore have no specific choice for their post-graduation field.^6^ Sanfery mentioned that clerkship done in the relevant fields play an important role this selection.^10^

Another trend seen in our institutes is the increasing number of foreign nationals coming to our country for higher education. This trend is also seen in by Akhund S in a study conducted on International students from outside Pakistan participated in the survey and 31% opted for surgery as a career choice.^14^

Family members attitudes plays an important role in which direction an individual may go in life regarding career choice, higher education, and personal life decision. It has been seen by Sadaf et al that in the 3^rd^ & 4^th^ year MBBS majority of the students are not sure which career path they may take.^15^ In our study 38% had a family member as a doctors which may be the driving force in their selection. The overall p-values were significant our study 0.016.

In our study we focused on the final year and students doing House job/Internship for our survey. These years are crucial in the decision making process. Medicine and Allied was the most sought after specialty 58% followed by surgery and allied 34%. The reason for selecting any specialty 24.9% was reported as the number of working hours. This factor had highest priority for later better quality of personal life when in practice. This was followed by higher income and other family responsibilities as the next most common reason. In another study done by Rehman A et al also mentioned that financial consideration was important for 69.7% of participants while choosing a particular speciality.^9^ A study in Kenya reflected upon the trend for students opting for a particular specialty. Medicine, Surgery and Orthopedics were common among male students whereas females were more inclined towards pediatrics. Prestige among the society was the main reason for choice of the career in the masculine gender whereas in females it was quoted as ‘the ease to raise a family’.^16^ Also studies in Malawi and South Africa hinted a similar picture.^1^,^17^

The country of origin also is an important factor is selecting any given specialty, as the chances of an individual getting into highly competitive field such as surgery & allied may be difficult in some countries such as USA & Canada. So their candidates tend to apply in the residency in specialties which are easier to get into.^18^-^20^ This had a significant p-value of 0.006 in our study. In another study done by Dorsey ER et al mentioned a controllable life style as an important factor influencing the career choices of medical graduates.^21^ This factor was even more important when the students were choosing Surgery & Allied with a p-value of 0.005. The country of origin also was an important variable for non-clinical specialties (Such as pathology, radiology and Basic Science) with a p-value of 0.000.

Reduced working hours means a better quality of personal life. This was the most important reason for selecting medical & allied as a career choice. Most of the individuals choosing for Family medicine also had the same reason.^3^,^5^,^22^

Through the workload and working hours are more in Surgery & Allied but somehow the individuals choosing it have a higher degree of passion for this field and another attraction being the higher income of surgeons.^18^ Another reason could be the first clinical experience of medical graduates.^23^-^24^

### Limitations

A larger sample size could help in increasing the power of the study. A follow up study should be done to see whether these individuals are satisfied with their choices and then changes should be made in the National Health Policies.

## CONCLUSION

The working hours followed by passion for the chosen field were the important reasons for selecting any specialty. The next most important reason was higher income and other family responsibilities of an individual. The ladies seem to be optioning more for the fields with a controllable life style.

### Authors’ Contribution

***SZ:*** Conceived, designed and did statistical analysis & manuscript writing.

***MA & MS:*** Data collection & editing of manuscript.

***SM:*** Review and final approval of manuscript.
